# Exploring the Neuroprotective Effects of Spirulina platensis: Insights Into Hemorrhagic Volume and Histological Outcomes

**DOI:** 10.7759/cureus.42078

**Published:** 2023-07-18

**Authors:** Timoteo Almeida, Gregori Manfroi, Stephanya Silva, Pamella Beggiora, Daniela Schwingel, Telma E Bertolin

**Affiliations:** 1 Department of Radiation Oncology, University of Miami, Miami, USA; 2 Department of Neurosurgery, University of Miami, Miami, USA; 3 Department of Neurosurgery, Hospital Santa Marcelina, São Paulo, BRA; 4 Department of Morphology and Pathology, Federal University of São Carlos, São Carlos, BRA; 5 Department of Surgery and Anatomy, Ribeirão Preto School of Medicine, University of São Paulo, Ribeirão Preto, BRA; 6 Department of Pathology, Faculdade Meridional School of Medicine, Passo Fundo, BRA; 7 Graduate Program in Food Science and Technology, University of Passo Fundo, Passo Fundo, BRA

**Keywords:** apoptosis, stroke, cerebral hemorrhage, neuroprotection, spirulina platensis

## Abstract

Background

Hemorrhagic events can result in significant neurological damage, and identifying effective strategies for neuroprotection is crucial. Several studies have directed their attention to the alterations in perilesional parenchymal tissue. These investigations have sought to modify ischemic and metabolic changes by utilizing potential neuroprotective agents and to develop strategies that effectively mitigate secondary perilesional neuronal damage. By gaining a deeper understanding of its mechanisms and efficacy, *Spirulina platensis* can emerge as a promising therapeutic intervention for various neurological disorders.

Methodology

This controlled and blinded experimental study was conducted on adult male Wistar rats. The rats were divided into the treatment group, which received *Spirulina platensis* extract for 30 days before the hemorrhagic event, and the control group, where all animals underwent the same experimental hemorrhage model using collagenase. Each group was divided into the following three subgroups based on the sacrifice time: six hours, 24 hours, and 30 days. The brain section with the largest hemorrhage volume was selected for histological analysis. The number of viable neurons was analyzed in the perilesional zone and the cortical fields along the puncture trajectory. Neurofunctional evaluations were conducted on animals sacrificed 15 and 30 days after the procedure.

Results

Initial analysis showed no significant difference in viable neurons between groups (p = 0.63). Still, after 24 hours, the treatment group had a significantly higher number of viable neurons per peripheral fields (18.5) compared to the control group (13.4; p < 0.05). Neurofunctional tests at 15 days indicated a trend toward significance in absolute discrimination (p = 0.054), with the control group showing higher mean values (5.5, SD = 3.1) than the treatment group (-1, SD = 5.1). The discrimination index exhibited a significant difference (p < 0.01), with higher mean values in the control group (0.59, SD = 0.34) compared to the treatment group (-0.05, SD = 0.21). No significant differences were found in other neurofunctional parameters at this time point. At 30 days, no significant differences were observed in absolute discrimination, discrimination index, contralateral paw elevation, rearing time, and wire hanging time test (p > 0.1); however, the treatment group presented a better motor performance in the open field test (14.2, SD = 9.02) compared to the control group (5.25, SD = 2.06), approaching significance (p = 0.06).

Conclusions

The group treated with *Spirulina platensis* demonstrated significantly more viable neurons in the perilesional fields 24 hours after the induced hemorrhage. The treatment group also had a relatively better motor performance in the open field test 30 days after the hemorrhage (p = 0.06). These findings suggest a potential neuroprotection effect and warrant further investigations to explore the effects of *Spirulina platensis* and its active component phycocyanin in acute neurological conditions.

## Introduction

Cerebral hemorrhage significantly contributes to stroke incidence, accounting for approximately 15% of all cases [1]. Systemic arterial hypertension stands out as the primary risk factor [2]. Spontaneous cerebral hemorrhage, especially common among elderly patients, is associated with high morbidity and mortality rates. Approximately 50% of patients do not survive, and up to 80% of survivors experience disability six months after the hemorrhagic event [3].

Considering the significant impact of spontaneous cerebral hemorrhage and the limited success of current standard-of-care treatments aimed at best medical management and early evacuation of the hematoma in improving functional outcomes, several studies have focused on the changes in the surrounding parenchymal tissue after acute neuronal injury (e.g., after ischemic or hemorrhagic stroke). The ultimate goal is to discover strategies to modify the ischemic and metabolic tissue alterations by employing neuroprotective agents with antioxidant, anti-inflammatory, anti-excitatory, immunomodulatory, iron-chelating, or gene therapy actions [4]. Iron release in the perilesional tissue primarily results from hemoglobin degradation and is responsible for the delayed edema observed in spontaneous cerebral hemorrhage, often leading to fatal outcomes [5-7]. Iron toxicity to the brain parenchyma mainly occurs through direct cellular damage and the production of reactive oxygen species [8]. Prior studies have demonstrated neuroprotective properties by reducing iron-mediated perilesional edema [9,10], the inflammatory response, or the secondary perilesional neuronal death following the primary hemorrhagic injury [9,11-15].

*Spirulina platensis*, a microalga with a high protein content (approximately 70% of its dry weight), has gained attention as a dietary supplement [16]. The potential neuroprotective effects of *Spirulina platensis* can be attributed to its active component, phycocyanin. Recent experimental studies have highlighted the clinical effects of *Spirulina platensis*, particularly its antioxidant, anti-inflammatory, and iron-chelating properties [17,18]. Moreover, phycocyanin exerts anti-apoptotic effects by modulating apoptotic pathways, promoting the expression of anti-apoptotic proteins, and inhibiting pro-apoptotic protein activation. These mechanisms prevent neuronal apoptosis and enhance neuronal survival. This study aims to investigate the effect of pretreatment with *Spirulina platensis* on cerebral hemorrhage in an experimental rat model.

## Materials and methods

Animals

This study was designed as a controlled and blinded experiment in rats. A total of 33 male Wistar rats weighing between 300 and 350 g and approximately three months old were used. The animals were kept in a climate-controlled environment at a temperature of 21 (±1)°C, with a 12-hour light-dark cycle, and had access to water and food ad libitum.

The animals were divided into treatment and control groups and further subdivided according to the time of euthanasia (six hours, 24 hours, or 30 days after hemorrhage). All rats in the treatment group received nutritional supplementation with *Spirulina platensis* via oral gavage, with a dosage of 1.67 × 10^-2^ g diluted in 2 mL of distilled water, starting 30 days before the procedure. This dosage was chosen based on the recommendation of the Food and Drug Administration for a daily intake of 3 g for a 60 kg adult.

The animal experiments conducted in this study were performed following the ethical guidelines and regulations set forth by the U.K. Animals (Scientific Procedures) Act, 1986 and associated guidelines, EU Directive 2010/63/EU for animal experiments, and the National Institutes of Health guide for the care and use of Laboratory animals (NIH Publications No. 8023, revised 1978). Adult male rats weighing between 300 and 350 g were used in the experiments. All efforts were made to ensure the humane treatment and handling of the animals. The sex of the animals was controlled to avoid confounding bias from intergender physiological and behavioral factors. Furthermore, the ARRIVE (Animal Research: Reporting of In Vivo Experiments) guidelines were followed to enhance the transparency and reproducibility of the research. These measures were taken to ensure compliance with the guidelines and regulations and to uphold the highest standards of animal welfare and ethical conduct throughout the study. The experiment was approved by the University of Passo Fundo Ethics Committee on Animal Use (protocol number: 0222014).

Cerebral hemorrhage model

We used an experimental cerebral hemorrhage model by injecting 0.5 U of collagenase VII (Sigma Chemical, USA) diluted in 2 µL of 0.9% saline solution at the level of the right putamen. All animals were subjected to intramuscular anesthesia with Zoletil 50® at a dose of 1 mL/kg and maintained under spontaneous ventilation with ambient air throughout the procedure. After anesthesia, trichotomy and scalp antiseptic were performed using germicidal chlorhexidine. Collagenase was injected using a Hamilton® 700 series syringe and guided by stereotactic points 3 mm lateral to the sagittal suture, over the coronal suture, at a depth of 6 mm, and with a 90-degree angle in relation to the sagittal suture. After the solution was injected, the needle was kept in place for two minutes to prevent additional bleeding from the abrupt removal, as previously described in stereotactic studies [19,20].

Neurofunctional assessment

The neurofunctional assessment was conducted in a quiet room with low lighting at 15 and 30 days after the induced hemorrhage and consisted of the below previously validated tests [21].

Open Field Test

The apparatus for this assessment consisted of a wooden box measuring 50 × 50 cm, surrounded by high walls of 40 cm and a transparent glass front wall, allowing the observer’s observation. The floor of the open field was divided into 12 equal areas by black lines. During the experiment, each rat was carefully placed inside the box, facing the rear wall, and remained inside for five minutes for free exploration. The recorded variables were the number of crossings over the black lines and the rearing behavior of the animal.

Forelimb Grip Strength Test

The time until the structure, consisting of a 2 mm diameter wire measuring 50 cm in length and placed at a height of 40 cm, fell was measured. A soft sponge was placed between the two supports to protect the animals.

Object Recognition Test

The task involved a training session in which the animals were exposed to an open field measuring 50 × 50 cm, made of wood, with a glass front, for 20 minutes. Two objects that differed in color, height, shape, and texture were placed equidistantly in the open field, attached to the base. After 24 hours, one of the objects was replaced with a different shape and color, and the animals were placed in the box for five minutes for free exploration. The objects and the open field were cleaned with 70% ethanol after each session to remove olfactory cues. The exploration time of each object in seconds was calculated, and the percentage of time spent exploring the new object was calculated to evaluate long-term memory.

Histological analysis

**Figure 1:**
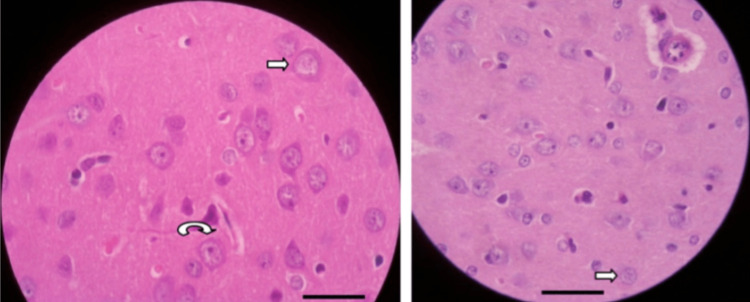
Rat brain (hematoxylin and eosin 400×). Scale bar = 50 µm. Perilesional zone showing cells in various stages of cellular injury. Straight arrows, chromatolysis. Curved arrow, cellular swelling.

All rats were euthanized by decapitation to avoid ischemic changes due to cerebral hypoperfusion, and the brains were immediately immersed in a 4% formalin solution. Subsequently, the brains were transversely sectioned with a thickness of 1 mm and embedded in paraffin. Histological analysis was performed on the brain section with the largest hemorrhagic area using hematoxylin-eosin and Prussian blue stains. Ten perilesional fields and three cortical fields in the path of the cerebral puncture were studied at a magnification of 400×. The number of viable neurons was counted using morphological criteria, as previously described (Figure 1).

## Results

The hemorrhagic volume was similar between the groups, with an average of 3.89 × 10^-3^ mL (SD = 1.77 × 10^-3^ mL) in the treatment group and 5.19 × 10^-3^ mL (SD = 3.62 × 10^-3^ mL) in the control group (p = 0.3).

**Figure 2:**
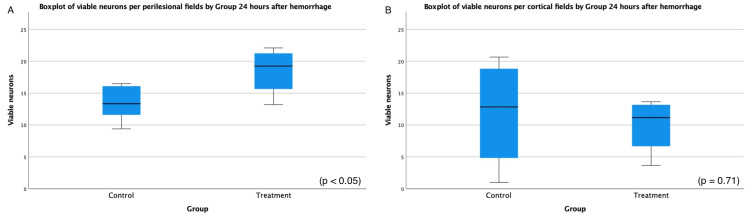
Boxplot of the number of viable neurons per perilesional (A) and cortical (B) fields 24 hours after hemorrhagic injury.

The number of viable neurons was analyzed six and 24 hours after the hemorrhage. Initially, there was no significant difference in the number of viable neurons between the two groups (p = 0.63). After 24 hours, there was a significantly higher number of viable neurons per peripheral field in the treatment group (18.5) compared to the control group (13.4; p < 0.05). The mean difference in the number of viable neurons per peripheral field in the control group was -5.07 ± 2.11 (range = -9.92 to -0.21), as determined by a t-test with a value of -2.41 and degree of freedom (DF) of 8. There was no significant difference in the number of viable neurons per field in the cortex between the two groups (Figure 2).

At the 15-day mark after hemorrhage, the neurofunctional tests revealed interesting findings. The absolute discrimination test showed a higher mean in the control group (5.5, SD = 3.1) than in the treatment group (-1, SD = 5.1) with a trend toward significance (p = 0.054). Furthermore, the discrimination index exhibited a higher mean in the control group (0.59, SD = 0.34) than in the treatment group (-0.05, SD = 0.21) with a statistically significant difference (p < 0.01). However, no significant differences were observed in other neurofunctional parameters at this time point. The contralateral paw elevation, rearing time, open field motor evaluation, and wire hanging time test did not exhibit statistically significant changes, with p-values of 0.67, 0.25, 0.82, and 0.41, respectively.

**Figure 3:**
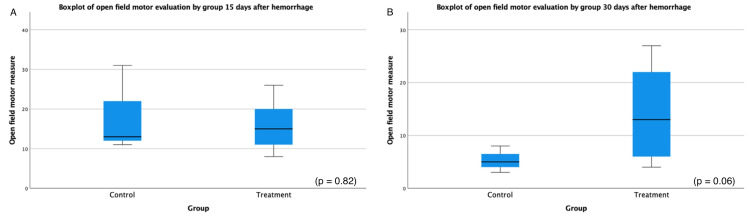
Boxplot of the open field motor test results at 15 (A) and 30 days (B) after induced intracerebral hemorrhage.

Moving to the 30-day assessment following the hemorrhage, the neurofunctional tests demonstrated less pronounced effects. There were no significant differences observed in absolute discrimination (p = 0.93) and discrimination index (p = 0.46). Similarly, no significant differences were found in contralateral paw elevation (p = 0.40), rearing time (p = 0.38), and wire hanging time test (p = 0.1) at the 30-day mark. However, the open field motor evaluation test exhibited a higher mean in the treatment group (14.2, SD = 9.02) compared to the control group (5.25, SD = 2.06) with a trend toward significance (p = 0.06), indicating a possible impact on motor performance. The mean difference in the open field motor evaluation measure in the control group was -8.92 ± 3.85 (range = -18.4 to 0.54), as determined by a t-test with a value of -8.92 and DF of 5.76 (Figure 3). Table 1 describes the independent-sample t-test results.

**Table 1:** Independent-sample t-test results. *: p < 0.05; **: p < 0.01.

		t-value	DF	Mean difference	Standard error	95% CI		Two-sided p-value
						Lower	Upper	
Hemorrhagic volume								
	6 hours after hemorrhage	-1.70	9	-2.27	1.34	-5.3	0.76	0.12
	24 hours after hemorrhage	-1.83	10	-0.35	1.93	-4.66	3.95	0.86
Number of viable neurons								
	Per perilesional field, 6 hours after hemorrhage	-0.49	10	-1.23	2.51	-6.84	4.37	0.63
	Per perilesional field, 24 hours after hemorrhage	-2.41	8	-5.07	2.11	-9.92	-0.21	0.043*
	Per cortical field, 6 hours after hemorrhage	0.13	8	0.42	3.13	-6.79	7.62	0.90
	Per cortical field, 24 hours after hemorrhage	0.39	6	1.92	4.94	-10.17	14	0.71
Neurofunctional tests								
	Absolute discrimination 15 days after hemorrhage	2.26	8	6.5	2.88	-0.14	13.14	0.054
	Absolute discrimination 30 days after hemorrhage	-0.09	8	-0.17	1.82	-4.58	4.24	0.93
	Discrimination index 15 days after hemorrhage	3.77	8	0.64	0.17	0.25	1.03	0.005**
	Discrimination index 30 days after hemorrhage	-0.78	8	-0.12	0.16	-0.49	0.21	0.46
	Contralateral paw elevation 15 days after hemorrhage	0.44	8	3.01	6.89	-12.88	18.91	0.67
	Contralateral paw elevation 30 days after hemorrhage	0.88	8	10.85	12.29	-17.49	39.19	0.40
	Rearing time 15 days after hemorrhage	-1.24	8	-5.92	4.76	-16.9	5.07	0.25
	Rearing time 30 days after hemorrhage	-0.94	8	-2.67	2.85	-9.24	3.9	0.38
	Open field 15 days after hemorrhage	0.23	8	1.17	4.99	-10.35	12.68	0.82
	Open field 30 days after hemorrhage	-2.33	5.76	-8.92	3.82	-18.37	0.54	0.06
	Wire hanging time 15 days after hemorrhage	0.18	8	2.08	11.61	-24.68	28.85	0.86
	Wire hanging time 30 days after hemorrhage	2.25	3.17	21.67	9.64	-8.12	51.45	0.11

## Discussion

Several studies have demonstrated the clinical activities of *Spirulina platensis* in experimental models, highlighting its antioxidant, anti-inflammatory, iron-chelating, and immunomodulatory effects. Phycocyanin-C, the main protein found in *Spirulina platensis*, has been identified to be responsible for these effects in previous studies [18,22-27].

Bermejo-Bescós et al. [17] demonstrated that *Spirulina platensis* extract or phycocyanin-C has the potential to reduce the production of hydroxyl and peroxyl radicals, inhibit lipid peroxidation, and possess iron-chelating activity. They have been indicated as potential treatments for neurodegenerative diseases due to the toxic action of iron in the pathogenesis of these diseases [22].

Similar to previously described studies in ischemia stroke and reperfusion treated prophylactically or therapeutically with phycocyanin-C, which showed reduced neuronal death in the hippocampus [28], we found a significantly higher number of viable neurons in the perilesional areas 24 hours after the hemorrhagic event. We believe that this is a result of the neuroprotective effects of *Spirulina platensis*, likely due to its active component phycocyanin, in preventing secondary injury due to its antioxidant and anti-inflammatory properties and reducing neuronal apoptosis.

It is important to observe that although no statistical difference in hemorrhagic volume was seen between the two groups, the treatment group presented a slightly higher volume at six hours. This difference can be related to the data presented by Kamble et al. [29] who reported that *Spirulina platensis* presented anticoagulant activity in vitro. The anticoagulant activity was also documented with the polysaccharide extract derived from the microalga *Arthrospira platensis* [30]. If this property is confirmed to be present in vivo, it can lead to a larger initial hematoma volume. The mildly higher hematoma volume in the treatment group can also be responsible for the worse results in the novel object exploration test 15 days after hemorrhage, possibly surpassing the potential antioxidant or anti-inflammatory effects of *Spirulina platensis* treatment at that time. One hypothesis is that the worse initial results in the new object exploration test could be secondary to the mass effect caused by the larger hemorrhagic volume in the treatment group. In contrast, the late improvement in viable neurons and motor performance in the treatment group are likely secondary to the neuroprotective effect of *Spirulina platensis*, leading to a lesser perilesional injury response. These findings align with the antioxidant and anti-inflammatory effects attributed to *Spirulina platensis*, which may contribute to reduced perilesional injury response.

The neuroprotective properties of *Spirulina platensis* have also been investigated in other acute neurological injuries, such as ischemic stroke. As discussed above, previous studies have demonstrated the antioxidant and anti-inflammatory effects of *Spirulina platensis* and its components, which can be beneficial in mitigating the damage caused by an ischemic stroke. Given the diverse mechanisms and pathophysiology underlying different neurological injuries, further investigation is warranted to fully explore the potential of *Spirulina platensis* as a neuroprotective agent. Additional studies focusing specifically on ischemic stroke models can shed light on its efficacy in this context. By elucidating the underlying mechanisms and optimizing treatment protocols, *Spirulina platensis* may hold promise as a therapeutic intervention for neuroprotection in ischemic stroke and other neurological conditions.

Future studies should also investigate the potential anticoagulant effect of phycocyanin extract derived from *Spirulina platensis*. Given the mildly higher intracerebral hemorrhage volume observed in the treatment group in this study, it is crucial to elucidate the underlying mechanisms and determine whether phycocyanin or other active components of *Spirulina platensis* possesses anticoagulant properties in vivo. Understanding the anticoagulant properties of phycocyanin will provide valuable insights into its safety profile and aid in refining its potential therapeutic application.

It is important to address some limitations of this study. First, the study was conducted using an experimental model, which may not fully represent the complexities of the neurological injury following spontaneous intracerebral hemorrhage. Second, it is worth noting that only male rats were used in this study to increase homogeneity between the groups, potentially introducing gender-specific biases.

## Conclusions

The better late-stage histological and motor outcomes in the treatment group, indicating a potential reduction in secondary perilesional neurological damage, favor a neuroprotective effect of *Spirulina platensis* in this experimental model in rats. This result is likely secondary to its antioxidant and anti-inflammatory properties. Understanding the mechanisms underlying the beneficial effects of *Spirulina platensis* in reducing neuronal injury and inflammation can pave the way for the development of novel therapeutic approaches for various neurological disorders. Future studies focusing on different injury models and elucidating the specific pathways involved will be crucial in thoroughly assessing the therapeutic potential of *Spirulina platensis* as a neuroprotective agent.
